# Clinical and Inflammatory Determinants of Heart Failure Severity Following Myocardial Infarction: Implications for Post-Infarction Care

**DOI:** 10.3390/jcdd13050197

**Published:** 2026-05-02

**Authors:** Alexandra Manuela Buzle, Priscilla Matache, Mădălina Ioana Moisi, Corina Cinezan, Marc Cristian Ghitea, Evelin Claudia Ghitea, Timea Claudia Ghitea, Ioana Adriana Ardelean, Marius Rus, Roxana Daniela Brata, Mircea Ioachim Popescu

**Affiliations:** 1 Department of Medical Disciplines, Faculty of Medicine and Pharmacy, University of Oradea, 410073 Oradea, Romania; buzle.alexandramanuela@student.uoradea.ro (A.M.B.); priscilla_pasc@uoradea.ro (P.M.); ccinezan@uoradea.ro (C.C.); rusmariusr@yahoo.com (M.R.); 2 Clinical County Emergency Hospital Bihor, 410169 Oradea, Romania; moisi.madalina.ioana@didactic.uoradea.ro (M.I.M.); adriana_toadere@yahoo.com (I.A.A.); 3 Department of Preclinical Discipline, Faculty of Medicine and Pharmacy, University of Oradea, 410068 Oradea, Romania; 4 Faculty of Medicine and Pharmacy, University of Oradea, 410068 Oradea, Romania; ghitea.marccristian@student.uoradea.ro (M.C.G.); ghitea.evelinclaudia@student.uoradea.ro (E.C.G.); 5 Pharmacy Department, Faculty of Medicine and Pharmacy, University of Oradea, 410068 Oradea, Romania; 6 Department of Clinical Discipline, Faculty of Medicine and Pharmacy, University of Oradea, 410068 Oradea, Romania; procardia_oradea@yahoo.com

**Keywords:** myocardial infarction, post-infarction heart failure, systemic inflammation, C-reactive protein (CRP), NT-proBNP

## Abstract

Background: Post-infarction heart failure (HF) remains a major contributor to morbidity and mortality despite advances in reperfusion and pharmacological management. However, the combined influence of clinical background, myocardial injury, neuro-hormonal activation, and angiographic disease on HF severity is not fully defined. Methods: We retrospectively analyzed 181 patients with confirmed myocardial infarction treated in a tertiary cardiology center. Demographics, cardiovascular risk factors, prior chronic HF, inflammatory markers (CRP, fibrinogen, ESR, leukocyte indices), and high-sensitivity troponin (hs-Tn) were measured at admission (pre-intervention), immediately after percutaneous coronary intervention (PCI), and at 48 h, angiographic lesion distributions were collected. HF severity was graded on a five-level scale and further dichotomized as no/mild HF (grade 0–1) versus moderate–severe HF (grade ≥ 2). Group comparisons and multivariable logistic regression were used to identify independent determinants of severe HF. Results: Moderate–severe HF occurred in 42.5% of patients (77/181). Compared to HF 0–1, the HF ≥ 2 group was older (64.0 vs. 60.5 years, *p* = 0.042) and exhibited substantially higher systemic inflammation (CRP 41.5 vs. 9.75 mg/L, *p* < 0.001; fibrinogen 435 vs. 346 mg/dL, *p* = 0.0002; ESR 28 vs. 18 mm/h, *p* = 0.0004). hs-Tn levels and NT-proBNP were significantly elevated in HF ≥ 2 (NT-proBNP 3449 vs. 1243 pg/mL, *p* = 0.0003), while left ventricular ejection fraction was reduced. Prior HF increased the likelihood of HF ≥ 2 (54.5% vs. 33.7%, *p* = 0.0078), and conservative therapy was associated with adverse outcomes (87.5% vs. 40.5%, *p* = 0.0235). In multivariable analysis, NT-proBNP remained the only independent predictor of moderate–severe HF, while CRP showed a positive but non-significant trend after adjustment. Conclusions: Post-MI HF severity reflects the combined influence of myocardial injury, neurohormonal stress, and systemic inflammatory activation. However, in multivariable analysis, NT-proBNP emerged as the dominant independent predictor of moderate–severe HF, while CRP reflected an associated but non-independent inflammatory signal.

## 1. Introduction

Acute myocardial infarction (MI) remains a leading contributor to global cardiovascular morbidity and mortality. Despite substantial progress in primary angioplasty techniques and guideline-directed medical therapy, post-infarction heart failure (HF) continues to occur frequently and carries a major prognostic impact. Clinical evolution following reperfusion is heterogeneous: while some patients maintain stable ventricular function, others progress to congestion, systolic impairment, and adverse remodeling even after timely revascularization. This suggests that the transition from MI to HF is influenced by mechanisms that extend beyond coronary obstruction alone, involving structural injury, neurohormonal activation, and systemic inflammation [1,2,3,4].

Post-MI HF represents the clinical manifestation of a multifactorial process in which myocardial necrosis, coronary disease burden, pre-existing comorbidities, and inflammatory activation contribute interactively rather than independently. Several previous studies have evaluated isolated determinants, such as infarct size or inflammatory markers, but evidence integrating clinical, angiographic, biochemical, and functional variables into a unified risk framework remains limited. The observation that patients with comparable ischemic presentations may diverge widely in subsequent ventricular evolution highlights the need for a more comprehensive understanding of HF susceptibility [5,6,7].

In this context, identifying which components—myocardial injury, inflammatory activity, coronary anatomy, or neurohormonal response—carry the greatest weight in the development of HF may assist in refining risk stratification and intervention planning. A multiaxial evaluation combining these predictors has the potential to support earlier recognition of individuals vulnerable to maladaptive remodeling [8,9,10,11].

The present study investigates, in a real-world cohort of 181 patients with MI, the clinical, angiographic, and neurohormonal determinants associated with post-infarction HF severity. In contrast to previous work primarily focused on isolated markers or diastolic outcomes, this analysis takes an integrated view of post-MI physiology, aiming to delineate the interplay between inflammation, myocardial injury, and treatment strategy. Our objective is to clarify the biological drivers of HF progression and to provide a structured basis for improved prognostic assessment and individualized monitoring. In routine clinical practice, advanced molecular profiling is rarely available during the acute phase of myocardial infarction. Therefore, identifying clinically accessible biomarkers that reflect the integrated impact of injury, inflammation, and neurohormonal activation remains highly relevant. This study focuses on routinely measured parameters to support pragmatic post-infarction risk stratification.

## 2. Materials and Methods

### 2.1. Study Design and Population

This retrospective observational study was conducted at the Clinical County Emergency Hospital Bihor, Oradea, Bihor County, Romania, a tertiary-care cardiology center providing interventional management for acute coronary syndromes. Patient data were collected consecutively between January 2022 and December 2024. We conducted a study involving patients admitted with acute coronary syndrome (ACS) who underwent interventional management in a tertiary interventional cardiology center. A total of 181 patients were consecutively included based on the availability of complete clinical, laboratory, and echocardiographic records relevant to the study endpoints. Left ventricular ejection fraction (LVEF) was documented in all cases, with 73.7% presenting preserved systolic function (LVEF ≥ 50%). Patients with reduced EF (<45%) were not excluded. Sensitivity testing showed that stratification by LVEF did not alter the direction or magnitude of observed associations, although impaired systolic function was recognized as a potential confounder given its overlap with diastolic impairment.

Given the retrospective design, the present study was not intended to establish mechanistic causality. Rather, it aimed to identify clinically accessible markers that integrate myocardial injury, neurohormonal stress, and systemic inflammation in real-world post-infarction care.

Pharmacological therapy was not included in multivariable modeling due to incomplete retrospective documentation. However, all patients were managed according to contemporary guideline-directed care, and this study aimed to reflect real-world clinical conditions rather than controlled therapeutic exposure.

### 2.2. Inclusion and Exclusion Criteria

Inclusion criteria:

- Age ≥ 18 years.
- Availability of post-admission laboratory and echocardiographic data relevant to study outcomes.
- Acute myocardial infarction confirmed by coronary angiography, with either PCI-based or conservative in-hospital management depending on anatomical suitability and clinical indication.

Availability of post-intervention laboratory profile (complete blood count, leukocytes, neutrophils, CRP) and echocardiographic functional evaluation

Exclusion criteria:

- Active systemic infection at presentation or prior to admission.
- Known autoimmune, malignant, or hematologic disease.
- Failed or incomplete revascularization procedure.
- Missing key inflammatory, functional, or myocardial injury biomarkers.
- Chronic immunosuppressive therapy at the time of admission.

### 2.3. Data Collection

Clinical and paraclinical data were retrieved from the institutional electronic database (Medis®, v5.2, Bucharest, Romania) and original patient observation forms.

Recorded variables included:

- Demographics: age, sex, urban/rural residence.
- Metabolic comorbidities: hypertension, diabetes mellitus type 2, dyslipidemia, obesity (clinical diagnosis or treatment documentation).
- Lifestyle: smoking status and alcohol consumption.
- Inflammation-related parameters: leukocytes and neutrophils (baseline, post-PCI, and 48h), C-reactive protein (CRP) (mg/L), erythrocyte sedimentation rate (mm/h), fibrinogen (mg/dL).

CRP was measured 24–48 h after hospital admission, in the post-procedural period, together with routine post-intervention laboratory evaluation. Pre-PCI CRP values were not consistently available in the retrospective dataset.

Heart failure (HF) severity was classified using a five-level scale based on echocardiographic assessment of diastolic dysfunction according to ESC/EACVI recommendations.

- Cardiac biomarkers: high-sensitivity troponin (hs-Tn) pre-PCI, post-PCI, and 48h; NT-proBNP (pg/mL).
- Echocardiography: LVEF and diastolic function grading. Sensitivity analyses excluding patients with reduced LVEF (<45%) yielded similar directional associations, supporting the robustness of the primary findings.
- Risk scores: TIMI and Killip classification.
- Renal function: eGFR (used in stratified sensitivity analyses).

Hematology and inflammatory indices were processed using Sysmex XN-1000™ analyzers; biochemical panels via Cobas c501; cardiac biomarkers via electrochemiluminescence (ECLIA) using Cobas e601 (Roche Diagnostics, Mannheim, Germany). All post-PCI blood samples were collected fasting at 24–48 h. Data were cleaned, merged, and cross-validated using Microsoft Excel (version 2308) before statistical processing. 

Blood samples were collected using standardized venipuncture techniques and processed in the hospital’s certified central laboratory. Internal and external quality control procedures were applied according to institutional and manufacturer standards to ensure analytical reliability.

Medication history (ACEi, ARB, β-blockers, statins, diuretics) was incomplete and therefore excluded from modeling; this is acknowledged as a limitation.

### 2.4. Definition of Heart Failure Severity

Heart failure severity was classified using a study-specific five-level integrated grading system developed for retrospective clinical stratification in the acute post-infarction setting. This construct was not intended to represent an internationally validated HF classification, but rather to provide a pragmatic severity framework integrating bedside clinical findings and echocardiographic evidence of ventricular dysfunction or congestion.

Grades were defined as follows:

- Grade 0: no clinical signs or symptoms of heart failure; no pulmonary congestion; no peripheral edema; hemodynamic stability; no relevant echocardiographic evidence of elevated filling pressures or significant ventricular dysfunction.
- Grade 1: mild HF, defined by limited symptoms or mild congestion not requiring escalated HF-specific treatment; possible isolated echocardiographic evidence of impaired relaxation or borderline filling pressure elevation without overt clinical decompensation.
- Grade 2: moderate HF, defined by clinically evident congestion requiring intensified medical management (e.g., diuretics or vasodilator adjustment), with supportive echocardiographic abnormalities consistent with increased filling pressures and/or impaired ventricular compliance.
- Grade 3: severe HF, defined by marked pulmonary or systemic congestion, substantial functional limitation, and clear echocardiographic evidence of advanced diastolic dysfunction and/or significant ventricular systolic impairment, requiring intensive in-hospital management.
- Grade 4: advanced or unstable HF, defined by overt hemodynamic compromise, major congestion, or severe post-infarction cardiac dysfunction requiring urgent escalation of care.

For the primary analysis, grades 0–1 were grouped as no/mild HF and grades ≥ 2 as moderate–severe HF. Echocardiographic diastolic dysfunction grade according to ESC/EACVI recommendations was assessed separately and was not used as the sole determinant of clinical HF severity.

This five-level grading system was specifically developed for retrospective integrated clinical-echocardiographic stratification in the acute post-infarction setting and should not be interpreted as a formally validated replacement for Killip, NYHA, or AHA/ACC staging systems. Rather, it represents a study-specific severity framework designed to improve phenotypic characterization of post-infarction HF (Supplementary Table S2).

### 2.5. Assessment of Diastolic Function

Diastolic function was evaluated according to 2016 ESC/EACVI guidelines using four diagnostic parameters:

- Average E/e’ > 14.
- Septal e’ < 7 cm/s or lateral e’ < 10 cm/s.
- Left atrial volume index (LAVI) > 34 mL/m^2^.
- Tricuspid regurgitation (TR) velocity > 2.8 m/s.

Transthoracic echocardiography was performed using GE Vivid E9 with M5Sc-D transducer. Measurements were processed with EchoPAC v203 following standardized acquisition. E/e′ was averaged from septal and lateral annuli. LAVI was calculated using the biplane Simpson method. TR velocity was obtainable in 91% of patients; its absence did not alter grade assignment. Grading was performed independently by two certified cardiologists blinded to biomarker data; interobserver reproducibility was excellent (κ = 0.86). Collinearity diagnostics demonstrated acceptable independence among included biomarkers (all VIF values < 2), minimizing the risk of unstable coefficient estimation.

Patients with ≤1 abnormal criterion were assigned grade 0; grades 1–3 represented increasing severity per ESC algorithm.

### 2.6. Statistical Analysis

Data were analyzed using SPSS v30 (IBM, Armonk, NY, USA). Continuous variables were tested for distribution and reported as mean ± SD or median (IQR). Categorical data were expressed as number (percentage). Missing values < 5% were handled by complete-case analysis.

The primary endpoint of this study was clinical post-infarction heart failure (HF) severity, dichotomized as no/mild HF (grades 0–1) versus moderate–severe HF (grade ≥ 2). This dichotomized HF outcome served as the dependent variable in the primary multivariable analysis.

Multivariable logistic regression was performed to identify independent clinical and biochemical predictors of moderate–severe HF (HF ≥ 2). Candidate variables were selected based on biological plausibility and statistical significance in univariable analyses.

Echocardiographic diastolic dysfunction grade (0–3) was analyzed separately as a secondary outcome using ordinal logistic regression, in order to explore structural–functional correlates of HF severity.

Multicollinearity was assessed using variance inflation factor (VIF) analysis, with VIF < 2 considered acceptable. Model calibration and goodness-of-fit were evaluated using the Hosmer–Lemeshow test.

Variables were entered into multivariable models using a theory-driven forced-entry approach, prioritizing biological relevance over automated selection procedures.

### 2.7. Ethical Statement

This study was conducted in accordance with the Declaration of Helsinki and approved by the Institutional Review Board of the University of Oradea (number of approval 2379 from 21 January 2025).

## 3. Results

### 3.1. Clinical Axis and Comorbidity Profile

A total of 181 patients with myocardial infarction were included in the analysis, with a mean age of 62.8 ± 11.2 years. Males represented 65.7% of the cohort, while females accounted for 34.3%. No significant sex-related differences were observed between patients with HF 0–1 and those with HF ≥ 2 (36.5% vs 31.2%, respectively). Patients who progressed to HF ≥ 2 were modestly but significantly older compared with those classified as HF 0–1 (median 64.0 vs. 60.5 years, *p* = 0.041), suggesting an age-related reduction in myocardial functional reserve and recovery capacity after ischemic injury. The cohort included 120 patients with STEMI (66.3%) and 61 with NSTEMI (33.7%). No significant difference in infarct subtype distribution was observed between patients with HF 0–1 and HF ≥ 2 (66.3% vs. 66.2%, *p* = 0.99).

A small subgroup (n = 8) received conservative management and showed higher HF severity; however, this finding should be interpreted cautiously due to the limited sample size.

Traditional cardiometabolic comorbidities—including hypertension, type 2 diabetes mellitus, dyslipidemia, obesity, and active smoking—were highly prevalent overall, but none of them significantly differentiated HF severity (all *p* > 0.05). 

Conversely, pre-existing chronic heart failure was a strong predictor of adverse evolution. Among patients with documented history of HF, 54.5% developed moderate–severe HF after MI, compared with 33.7% of those without prior HF (*p* = 0.0078). This association highlights the role of impaired baseline myocardial reserve and pre-established remodeling as determinants of subsequent decompensation (Table 1).

### 3.2. Myocardial Injury and Neurohormonal Axis

In adjusted multivariable analysis, NT-proBNP remained the only independent predictor of moderate–severe HF, while CRP showed only a non-significant positive trend. 

Markers of myocardial injury and ventricular stress demonstrated strong and consistent associations with post-infarction HF severity. Patients in the HF ≥ 2 group exhibited significantly higher high-sensitivity troponin concentrations at all measurement times: pre-intervention (median 800 vs. 280 ng/L, *p* = 0.0015), post-procedural (median 41,254 vs. 25,079 ng/L, *p* = 0.030), and at 48 h (median 23,547 vs. 14,932 ng/L, *p* = 0.026). These differences indicate a greater extent of myocardial necrosis and cardiomyocyte membrane damage among patients who progressed to moderate–severe HF.

Neurohormonal activation followed a similar pattern. NT-proBNP levels were more than doubled in the HF ≥ 2 cohort when compared with HF 0–1 (median 3449 vs. 1243 pg/mL, *p* = 0.0003), reflecting increased wall stress, volume overload, and impaired ventricular compliance. Left ventricular ejection fraction was correspondingly lower in patients with HF ≥ 2 (median 40% vs. 49%, *p* = 0.001), supporting the link between structural injury, chamber dysfunction, and symptomatic deterioration.

**Figure 1:**
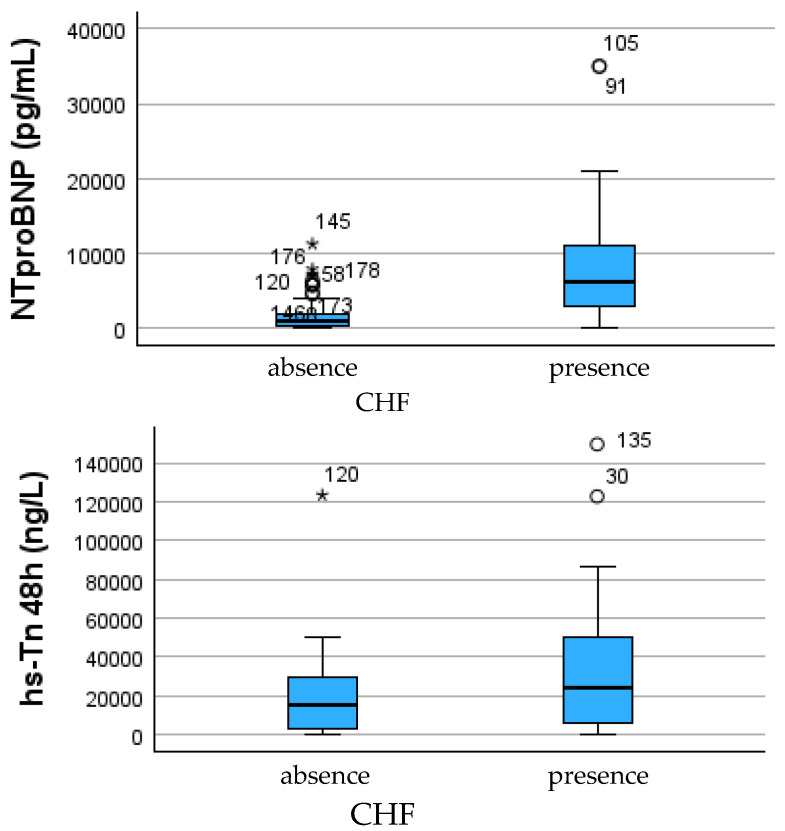
Distribution of NT-proBNP (**left**) and ultrasensitive troponin at 48 h (**right**) according to the severity of heart failure secondary to myocardial infarction. Patients with moderate–severe heart failure (HF ≥ 2) have significantly higher levels of both NT-proBNP and troponin compared with those with no or minimal heart failure (HF 0–1), suggesting an association between the magnitude of myocardial injury, wall stress, and clinical decompensation. * indicates extreme outliers, while ° denotes mild outliers, as defined by the boxplot convention (values exceeding 3 × IQR and 1.5–3 × IQR from the interquartile range, respectively).

Despite strong univariable associations for troponin, NT-proBNP, and LVEF, multivariable logistic regression identified NT-proBNP as the only independent predictor of HF ≥ 2, while CRP showed only a positive non-significant trend after adjustment. This emphasizes the central role of neurohormonal stress in post-infarction HF severity (Table 2 and Figure 1).

#### Sensitivity Analysis Including LVEF

A sensitivity analysis including LVEF as an additional covariate was performed to assess whether the association between CRP and HF severity persisted after adjustment for systolic function. After inclusion of LVEF, CRP did not retain statistical significance, whereas NT-proBNP remained independently associated with moderate–severe HF.

These findings indicate that while inflammatory activation accompanies HF severity, neurohormonal stress—as reflected by NT-proBNP—represents the primary independent determinant in adjusted models.

### 3.3. Angiographic and Therapeutic Axis

Coronary angiographic evaluation revealed multivessel disease in 50.3% of the cohort; however, the presence of two or more major vessel lesions did not significantly differentiate HF severity (*p* = 0.402). Although ischemic burden contributes to myocardial vulnerability, these findings suggest that anatomical extent of disease alone is insufficient to predict post-MI functional deterioration in the absence of accompanying inflammatory or neurohormonal escalation.

In contrast, management strategy showed a distinct impact on clinical outcome. Patients treated conservatively had a markedly higher prevalence of HF ≥ 2 compared with those undergoing percutaneous coronary intervention (87.5% vs. 40.5%, *p* = 0.0235). While the number of conservatively treated cases was small (n = 8), the magnitude of difference indicates that lack of revascularization may accelerate adverse remodeling and limit myocardial salvage capacity. These results are consistent with the concept that prompt reperfusion mitigates infarct expansion, preserves microvascular integrity, and reduces subsequent ventricular dysfunction. Given the very small number of conservatively treated patients (n = 8), this association should be interpreted as hypothesis-generating rather than causal.

**Figure 2:**
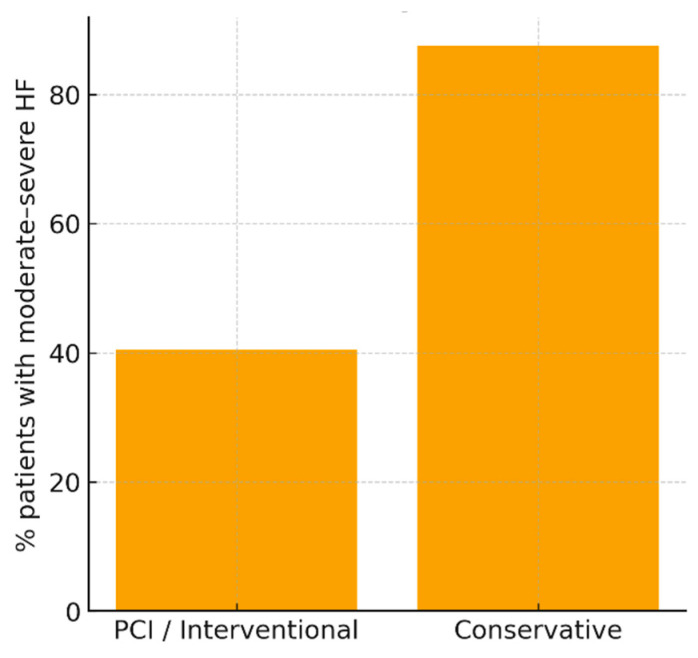
Proportion of patients with moderate–severe heart failure (HF ≥ 2) according to the therapeutic strategy applied. Conservative treatment is associated with a much higher prevalence of post-infarction dysfunction compared with coronary intervention (approximately 88% versus 40%), suggesting that revascularization probably reduces the risk of adverse ventricular remodeling.

The absence of significant association between multivessel involvement and HF severity, in conjunction with the strong therapeutic signal favoring intervention, supports the hypothesis that functional recovery depends more on myocardial salvage kinetics and the associated inflammatory response than on structural coronary burden alone (Table 3 and Figure 2).

### 3.4. Prognostic Scales and Integrated Risk Interpretation

Acute prognostic scales (Killip class, TIMI score, and myocardial blush grade—MBG) demonstrated visible distributional differences between HF 0–1 and HF ≥ 2 groups, although these trends did not reach independent statistical significance in multivariable modeling. Higher Killip and TIMI values, along with lower MBG reperfusion grade, were more frequently observed among patients who progressed to moderate–severe HF, suggesting that hemodynamic instability and suboptimal microvascular perfusion may contribute to post-ischemic vulnerability.

However, the lack of statistical independence for these scores compared with biochemical markers implies that acute clinical grading alone cannot fully capture remodeling risk. Inflammatory and neurohormonal responses appear to supersede hemodynamic scores in prognostic strength, positioning CRP and NT-proBNP as more dynamic indicators of myocardial stress, endothelial activation, and extracellular matrix turnover. This interpretation is consistent with the multivariable analysis, where CRP did not retain independent statistical significance.

**Figure 3:**
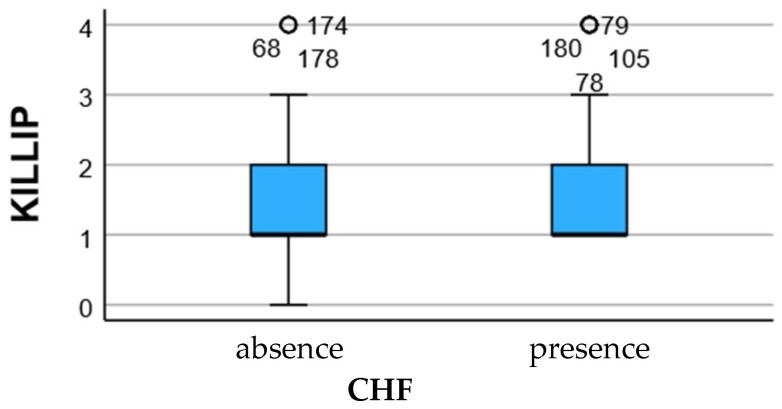

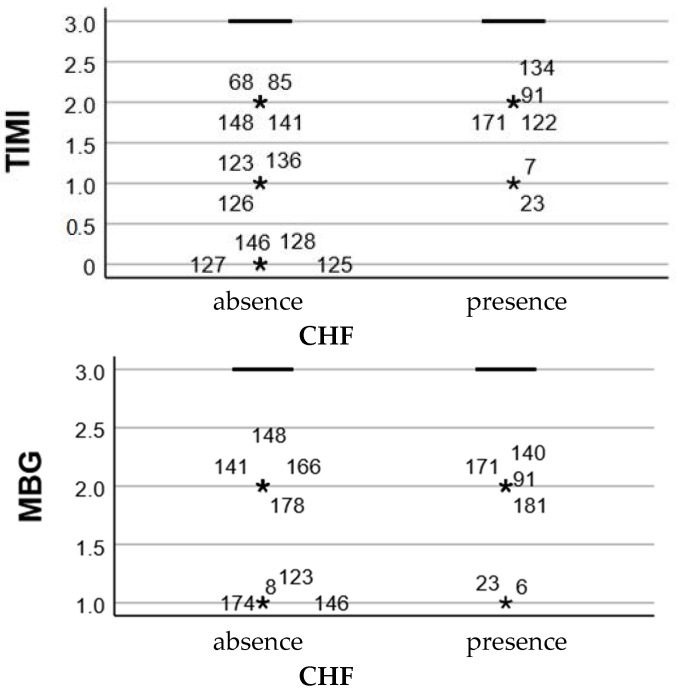
Distribution of acute severity scores (KILLIP, TIMI and MBG) according to the degree of heart failure secondary to myocardial infarction. Although a trend towards higher KILLIP and TIMI scores and lower MBG values is observed in the HF ≥ 2 group, these variations do not reach the independent statistical threshold, suggesting that acute hemodynamic deterioration does not fully explain the occurrence of severe HF, compared to the impact of inflammatory markers and tissue injury. * indicates extreme outliers, while ° denotes mild outliers, as defined by the boxplot convention (values exceeding 3 × IQR and 1.5–3 × IQR from the interquartile range, respectively).

Together, the findings across the clinical, biochemical, angiographic, and prognostic axes suggest that HF progression is best interpreted through an integrated model in which myocardial injury provides the substrate, coronary reperfusion modulates salvage, and inflammation appears to modulate the severity of the remodeling response without demonstrating independent predictive value after adjustment (Figure 3).

In multivariable logistic regression analysis, log-transformed NT-proBNP remained the only independent predictor of moderate–severe post-infarction heart failure (aOR 2.91, 95% CI 2.04–4.16, *p* < 0.001). CRP showed a positive association with HF severity; however, this did not retain conventional statistical significance after adjustment (aOR 1.28, 95% CI 0.97–1.68, *p* = 0.077). Age, prior chronic heart failure, and hs-troponin at 48 h were not independently associated with the outcome in the final model. The final multivariable model is presented in Table 4. Model discrimination was good (AUC = 0.86). Calibration assessed by the Hosmer–Lemeshow test was borderline (*p* = 0.047), suggesting acceptable but not optimal fit (Table 4).

#### Secondary Analysis: Diastolic Dysfunction, OR, Odds Ratio; CI, Confidence Interval (95%)

As a secondary analysis, ordinal logistic regression was performed using echocardiographic diastolic dysfunction grade (0–3) as the dependent variable. Increasing age was independently associated with more severe diastolic dysfunction (OR 1.04, 95% CI 1.01–1.07, *p* = 0.010), while higher LVEF showed a protective association (OR 0.96, 95% CI 0.92–1.00, *p* = 0.027). Neither CRP nor NT-proBNP retained statistical significance in this model (*p* > 0.05) (Table 5).

## 4. Discussion

### 4.1. Clinical Profile and Comorbidity Axis

In this study, the primary outcome was defined as clinical heart failure severity, while echocardiographic diastolic dysfunction grading was used as a secondary, supportive functional parameter. We found that age and prior chronic heart failure (HF) were associated with the development of moderate–severe post-infarction HF, although traditional cardiovascular risk factors such as hypertension, diabetes, obesity, dyslipidemia, and active smoking did not discriminate between clinical outcomes. This suggests that while cardiometabolic burden contributes to the long-term susceptibility to coronary artery disease, the immediate trajectory toward post-MI HF depends less on baseline risk load and more on myocardial resilience and inflammatory response during the acute phase. Patients with pre-existing HF were significantly more likely to progress to HF ≥ 2, indicating that structural and neurohormonal remodeling predating the infarction may reduce compensatory reserve and accelerate decompensation after ischemic injury [12,13].

### 4.2. Myocardial Injury and Neurohormonal Determinants

Troponin release and NT-proBNP levels were markedly higher in patients who developed moderate–severe HF, implicating both necrotic burden and subsequent ventricular wall stress as drivers of decompensation. This aligns with evidence showing that infarct size and neurohormonal activation remain independent clinical predictors of HF progression despite optimized acute reperfusion. Interestingly, after multivariable adjustment, NT-proBNP remained the only robust independent predictor. CRP appears to reflect the inflammatory burden associated with myocardial injury and ventricular dysfunction, but its association is attenuated after adjustment for systolic function. This highlights the role of systemic inflammation as a dynamic interface between tissue injury, extracellular matrix remodeling, and left ventricular dysfunction. Although inflammatory markers showed strong univariable associations, their effect was attenuated after multivariable adjustment, indicating that systemic inflammation reflects disease severity rather than acting as an independent determinant. In contrast, NT-proBNP consistently emerged as the dominant independent predictor, underscoring the central role of neurohormonal stress in post-infarction HF severity [14,15,16,17,18,19,20,21].

### 4.3. Angiographic Axis and Therapeutic Implications

Although multivessel disease was frequent in our cohort (~50%), angiographic burden was not independently associated with HF severity. This reinforces the concept that the quantity of obstructive lesions does not always reflect the biological complexity of infarction or the inflammatory milieu surrounding necrotic tissue. Conversely, the very high proportion of HF ≥ 2 among patients treated conservatively (~88%) highlights the clinical relevance of reperfusion timing and myocardial salvage. While conservative management applied to only a minority of patients, the outcome contrast with PCI-treated patients was striking, supporting early invasive revascularization as a key determinant of ventricular preservation and post-MI functional stability [22,23,24]. The observed association between conservative management and more severe HF is likely influenced by confounding by indication, as patients selected for non-interventional therapy may have presented with higher baseline risk, anatomical complexity, or contraindications to PCI. Accordingly, treatment strategy was not included in multivariable modeling, and no causal inference regarding therapeutic effects is implied.

### 4.4. Acute Prognostic Scales—Supportive but Not Definitive

Killip, TIMI, and MBG scores showed visual divergence between HF 0–1 and HF ≥ 2 groups, but these differences did not independently predict HF severity in multivariate testing. These findings suggest that prognostic scales retain utility for acute risk stratification but are insufficient for long-term functional outcome prediction when considered in isolation. Integrating biochemical markers—especially CRP as a clinically accessible indicator of inflammatory burden may complement prognostic assessment, despite not demonstrating independent predictive value in adjusted models [25,26,27,28,29].

### 4.5. Interpretation, Clinical Relevance, and Pathophysiological Integration

Our results indicate that the transition from myocardial infarction to heart failure is mediated by an interaction between myocardial injury, neurohormonal activation, and systemic inflammation, with the inflammatory component representing an associated biological response linked to myocardial injury and remodeling. Mechanistically, inflammatory mediators modulate post-ischemic fibroblast activation, collagen turnover, and matrix cross-linking—processes known to govern ventricular stiffness and progressive systolic impairment. Thus, CRP may function not only as a biomarker but also as a proxy measure of the remodeling cascade [30,31,32,33]. Although CRP is not a mechanistic mediator per se, its elevation reflects the magnitude of the systemic inflammatory response accompanying myocardial necrosis. In this context, CRP likely integrates upstream cytokine signaling, endothelial activation, and innate immune response, making it a clinically useful indicator of patients at higher risk for post-infarction heart failure.

CRP should be interpreted as a clinically accessible indicator of systemic inflammatory burden rather than a direct mechanistic mediator. Its elevation likely reflects upstream cytokine signaling and immune activation accompanying myocardial injury, making it useful for early clinical risk stratification rather than causal inference.

Because CRP was assessed in the early post-PCI window, its elevation should be interpreted as an integrated marker of acute infarct-related inflammation, tissue injury, and possibly procedure-associated inflammatory activation, rather than as a pure baseline inflammatory state. Our findings are consistent with the prior literature showing that elevated CRP levels in the acute phase of myocardial infarction are associated with worse short-term outcomes, including in-hospital heart failure, post-discharge HF, and mortality. Previous cohort studies and meta-analyses have demonstrated that CRP reflects both infarct-related inflammatory burden and adverse ventricular remodeling after AMI, particularly in patients undergoing PCI [34,35,36].

While the contribution of inflammation to post-infarction remodeling is well established, the novelty of the present study lies in its integrated, real-world evaluation of inflammatory burden, neurohormonal activation, angiographic strategy, and HF severity, using routinely available clinical parameters within a unified analytical framework. Rather than focusing on HF incidence alone, this study emphasizes HF severity as a clinically meaningful endpoint, with biomarker assessment performed within a defined early post-infarction window.

Importantly, this work diverges from previous research focused primarily on inflammation-driven diastolic dysfunction or post-interventional infection risk. Here, HF severity was examined within a broader pathobiological framework integrating angiography, neurohormonal load, comorbidities, and reperfusion strategy, offering a more comprehensive risk architecture.

### 4.6. Strengths and Limitations

The strengths of this study include a well-characterized cohort, simultaneous integration of clinical, biochemical, and angiographic dimensions, and stratification across a five-level HF severity scale. Limitations include the retrospective single-center nature, lack of longitudinal NT-proBNP tracking, and absence of cardiac MRI-based infarct quantification or fibrosis mapping. In addition, inflammatory markers beyond CRP, fibrinogen, and ESR were not available, which may underestimate the granularity of immune activation.

The present findings should not be interpreted as evidence of direct inflammatory causality, but rather as support for inflammation as a clinically relevant modifier of post-infarction heart failure severity.

#### Limitations and Future Perspectives

A major limitation is the absence of sufficiently complete medication data for robust adjustment. In post-MI patients, guideline-directed medical therapies such as ACE inhibitors/ARBs, beta-blockers, statins, mineralocorticoid receptor antagonists, and diuretics can substantially influence both HF progression and inflammatory biomarker profiles. Their exclusion from the multivariable model introduces the possibility of residual confounding and limits causal interpretation of the observed biomarker associations.

This work is retrospective and single-center, which may limit generalizability. Cardiac MRI and longitudinal NT-proBNP measurements were not available to further characterize fibrosis progression and neurohormonal trajectory. Inflammatory profiling was restricted to CRP, ESR, fibrinogen, and leukocyte indices, without cytokine-level granularity.

The inclusion of both STEMI and NSTEMI presentations reflects real-world acute MI practice but may contribute to clinical heterogeneity, given known differences in infarct size, inflammatory response, biomarker kinetics, and short-term HF risk.

The cohort included 181 patients with STEMI 120 (66.3%) and with NSTEMI 61 (33.7%), which may differ in infarct size, inflammatory response, biomarker kinetics, and short-term HF risk. Although this heterogeneity reflects real-world acute MI practice, infarct subtype may have acted as a residual confounder and should be more explicitly addressed in future prospective studies.

An additional limitation is the lack of consistently recorded data regarding time from symptom onset to reperfusion (door-to-balloon time), which may influence infarct size, biomarker kinetics, and early post-infarction HF development.

Pharmacological therapy was not included in multivariable modeling due to incomplete retrospective documentation; however, all patients were managed according to contemporary guideline-directed medical therapy, reflecting real-world clinical practice.

Although model discrimination and calibration were assessed using AUC and the Hosmer–Lemeshow test, more advanced evaluation of clinical utility, such as calibration plotting or decision curve analysis, would be more appropriately performed in larger prospective validation cohorts.

Future research should validate these findings in prospective, multicenter cohorts and incorporate molecular inflammatory markers, cardiac MRI-derived infarct size and extracellular volume, as well as follow-up echocardiography to map remodeling dynamics. Integration of CRP into combined biomarkers + imaging risk models may refine post-infarct prediction tools.

Despite these limitations, this study reflects routine cardiology practice and provides clinically actionable insights using widely available biomarkers.

## 5. Conclusions

Post-infarction heart failure severity appears to reflect the combined influence of myocardial injury, neurohormonal stress, and systemic inflammatory activation. NT-proBNP remained the most robust independent predictor of moderate–severe post-infarction HF, while the association of CRP was attenuated after adjustment for systolic function. However, because CRP was measured during the acute post-infarction phase, its elevation should be interpreted as an integrative marker of infarct-related inflammatory response and early remodeling burden rather than as evidence of an autonomous causal inflammatory pathway.

Inflammatory activity appears to act as an associated integrative signal of myocardial injury and remodeling rather than an independent driver, while NT-proBNP remains the most robust predictor of HF severity in adjusted analyses. CRP may assist clinicians in early identification of patients at risk for adverse remodeling, guiding closer monitoring and possibly anti-inflammatory therapeutic strategies.

## Figures and Tables

**Table 1:** Clinical characteristics and comorbidities by heart failure severity after myocardial infarction.

Variable	HF 0–1 (*n* = 104)	HF ≥ 2 (*n* = 77)	*p*-Value
Age (years), median (IQR)	60.5 (52.8–69.5)	64.0 (57.0–70.0)	0.041
Female sex, n (%)	38 (36.5%)	24 (31.2%)	0.460
HTN, %	58.8%	41.2%	0.79
Type II DM, %	41.6%	44.6%	0.83
Dyslipidemia, %	85.4%	41.8%	0.82
Obesity, %	48.1% vs 43.7%		0.88
Active smoker, %	38.6% vs 46.2%		0.38
Pre-existing CHF, %	33.7% vs 54.5%		0.0078

CHF, congestive heart failure; HTN, hypertension; DM, type II diabetes mellitus; IQR, interquartile range.

**Table 2:** Myocardial injury and neurohormonal biomarkers in patients with no/mild versus moderate–severe post-infarction heart failure.

Marker	CHF 0–1	CHF ≥ 2	*p*
hs-TN pre	280 (68–1082)	800 (278–2181)	0.0015
hs-TN post	25079 (5181–50.000)	41254 (13129–50.000)	0.030
hs-TN 48h	14932 (2583–31938)	23547 (6022–45827)	0.026
NT-proBNP	1242 (890–3818)	3449 (1602–8032)	0.0003

hs-Tn, high-sensitivity troponin; NT-proBNP, N-terminal pro-B-type natriuretic peptide; CHF, congestive heart failure.

**Table 3:** Angiographic profile and treatment strategy in relation to moderate–severe post-infarction heart failure.

Variable	% CHF ≥ 2	*p*
Multivascular disease	46.1%	0.402
Conservative treatment	87.5%	0.0235

CHF, congestive heart failure; multivascular disease = ≥2 major coronary vessels involved.

**Table 4:** Multivariable logistic regression for moderate–severe post-infarction HF.

Variable	Adjusted OR	95% CI	*p*-Value
log(CRP)	1.28	0.97–1.68	0.077
Age (per year)	1.01	0.97–1.04	0.683
Prior chronic HF	1.56	0.51–4.80	0.436
log(NT-proBNP)	2.91	2.04–4.16	<0.001
log(hs-Tn 48h)	0.98	0.85–1.13	0.768

**Table 5:** Ordinal logistic regression for diastolic dysfunction grade.

Variable	OR	95% CI	*p*-Value
Age (per year)	1.04	1.01–1.07	0.010
LVEF (per 1%)	0.96	0.92–1.00	0.027
log(CRP)	1.00	0.80–1.25	0.978
log(NT-proBNP)	1.21	0.96–1.53	0.102

## Data Availability

The datasets generated and analyzed during the current study are available from the corresponding author upon reasonable request and in accordance with institutional ethical regulations.

## Supplementary Materials

The following supporting information can be downloaded at: https://www.mdpi.com/article/10.3390/jcdd13050197/s1, Table S1: Complete univariable association analysis for moderate–severe post-infarction HF; Table S2: Study-specific heart failure severity grading criteria (grades 0–4).

## Abbreviations

The following abbreviations are used in this manuscript:

Abbreviation	Definition
MI	Myocardial Infarction
ACS	Acute Coronary Syndrome
HF	Heart Failure
Post-MI HF	Post-infarction Heart Failure
LVEF	Left Ventricular Ejection Fraction
PCI	Percutaneous Coronary Intervention
MVD	Multivessel Disease
CRP	C-Reactive Protein
ESR	Erythrocyte Sedimentation Rate
hs-Tn	High-sensitivity Troponin
NT-proBNP	N-terminal Pro-B-type Natriuretic Peptide
eGFR	Estimated Glomerular Filtration Rate
BMI	Body Mass Index
DM2/T2DM	Type 2 Diabetes Mellitus
HTN	Hypertension
MBG	Myocardial Blush Grade
TIMI	Thrombolysis in Myocardial Infarction score
TR	Tricuspid Regurgitation
LAVI	Left Atrial Volume Index
EchoPAC	Echocardiography Processing Software (GE Healthcare)
OR/aOR	(Adjusted) Odds Ratio
IQR	Interquartile Range
SD	Standard Deviation
