# Supplementary material for: Clinical and Inflammatory Determinants of Heart Failure Severity Following Myocardial Infarction: Implications for Post-Infarction Care
Source: J Cardiovasc Dev Dis. 2026 May 2;13(5):197. doi: 10.3390/jcdd13050197 (PMC13206775; doi:10.3390/jcdd13050197)
Supplement: Supplementary file 1 [file jcdd-13-00197-s001.zip › jcdd-4186158-supplementary.pdf]

Supplementary Table S1. Complete univariable association analysis for moderate–severe post-infarction HF

Variable	HF 0–1 (n=104)	HF ≥2 (n=77)	p-value
Age, median (IQR)	60.5 (52.8–69.5)	64.0 (57.0–70.0)	0.041
Female sex, n (%)	38 (36.5%)	24 (31.2%)	0.46
Hypertension, n (%)	58 (55.8%)	46 (59.7%)	0.61
Type 2 diabetes, n (%)	31 (29.8%)	28 (36.4%)	0.35
Dyslipidemia, n (%)	43 (41.3%)	35 (45.5%)	0.58
Obesity, n (%)	50 (48.1%)	34 (44.2%)	0.60
Active smoking, n (%)	40 (38.5%)	35 (45.5%)	0.35
Prior chronic HF, n (%)	11 (10.6%)	22 (28.6%)	0.003
STEMI, n (%)	69 (66.3%)	51 (66.2%)	0.99
NSTEMI, n (%)	35 (33.7%)	26 (33.8%)	0.99
hs-Tn 48 h, median (IQR)	2180 (1040–4110)	3470 (1810–6030)	0.012
NT-proBNP, median (IQR)	1242 (890–3818)	3449 (1602–8032)	<0.001
CRP, median (IQR)	18.4 (9.1–39.7)	33.6 (16.8–61.5)	0.006
LVEF, mean ± SD	53.8 ± 7.4	46.2 ± 8.1	<0.001
Multivessel disease, n (%)	47 (45.2%)	39 (50.6%)	0.47
TIMI ≤2, n (%)	19 (18.3%)	18 (23.4%)	0.40
MBG ≤2, n (%)	23 (22.1%)	20 (26.0%)	0.55
Conservative management, n (%)	2 (1.9%)	6 (7.8%)	0.048

Supplementary Table S2. Study-specific heart failure severity grading criteria (grades 0–4)

HF grade	Clinical criteria	Echocardiographic criteria	Approximate relationship with established scales
Grade 0	No symptoms or signs of heart failure; hemodynamically stable; no pulmonary rales, peripheral edema, or dyspnea	Normal or near-normal diastolic filling parameters; preserved systolic function when present	Killip I; NYHA I; AHA/ACC Stage A–B
Grade 1	Mild symptoms suggestive of early HF (exertional dyspnea, minimal congestion), without need for intensive HF-directed therapy	Mild diastolic dysfunction (impaired relaxation pattern), preserved or mildly reduced LVEF	Killip I–II; NYHA I–II; AHA/ACC Stage B
Grade 2	Moderate HF with clinically evident congestion requiring intensified treatment (diuretics/vasodilator adjustment); dyspnea and/or mild pulmonary congestion	Elevated filling pressures, moderate diastolic dysfunction, and/or mildly reduced LVEF	Killip II; NYHA II–III; AHA/ACC Stage C
Grade 3	Severe HF with marked pulmonary congestion, substantial functional limitation, orthopnea, or edema requiring intensive in-hospital management	Advanced diastolic dysfunction and/or significantly reduced LVEF with elevated filling pressures	Killip III; NYHA III–IV; AHA/ACC Stage C–D

Grade 4	Advanced or unstable HF with overt hemodynamic compromise, pulmonary edema, or cardiogenic shock requiring urgent escalation of care	Severe ventricular dysfunction with major systolic and/or diastolic impairment	Killip IV; NYHA IV; AHA/ACC Stage D
